# Catheter Ablation of Atrial Fibrillation in Chronic Heart Failure: A Contemporary Review

**DOI:** 10.19102/icrm.2017.080304

**Published:** 2017-03-15

**Authors:** Mario Matta, Fiorenzo Gaita, Matteo Anselmino

**Affiliations:** ^1^Division of Cardiology, Department of Medical Sciences, “Città della Salute e della Scienza” Hospital, University of Turin, Italy

**Keywords:** Atrial fibrillation, cardiomyopathy, catheter ablation, chronic heart failure

## Abstract

Catheter ablation of atrial fibrillation (AF) is a widely recommended treatment for patients presenting with symptomatic AF refractory to pharmacological treatment. AF ablation is also becoming a therapeutic option for patients with chronic heart failure (CHF), on top of optimal medical treatment, as AF is related to a higher risk of death, the worsening of symptoms, and the progression of CHF in this patient cohort. The present systematic review describes all published experiences concerning the use of AF catheter ablation among CHF patients and/or patients with structural cardiomyopathies, in an effort to summarize procedural safety and efficacy in this specific setting. Moreover, the effects of AF ablation on functional class and quality of life, as well as the different procedural protocols available, are presented and discussed, aiming to provide an evidence-based clinical perspective to optimize indication and tailor procedural characteristics and endpoints to patients affected by CHF referred for AF ablation.

## Introduction

Atrial fibrillation (AF) and chronic heart failure (CHF) are two modern epidemics that share many pathophysi-ological links, as demonstrated by their increasing prevalence, often in parallel, among the general population.^1–3^ In fact, CHF favors AF occurrence through an increase in left ventricular (LV) filling pressures, resulting in left atrial (LA) dilation and fibrosis. This structural remodeling is usually accompanied by electrical remodeling, as AF itself favors AF perpetuation.^4^ On the other hand, AF can increase the risk of developing LV dysfunction, which can result in CHF through the loss of atrial contraction, an uncontrolled heart rate with the presence of short and irregular cardiac cycles that can worsen mitral regurgitation.^5^ This may ultimately lead to impaired ventricular filling, contractility, and reduced cardiac output.^6^

AF relates to increased mortality in this population, and its treatment among patients with CHF plays a relevant role.^7^ Despite large randomized trials in which the use of rate control strategies resulted in non-inferior results with regards to rhythm control concerning hard end-points such as mortality and stroke, large observational cohorts described a beneficial effect for rhythm control, reporting longer survival rates and a reduced incidence of stroke and/or silent cerebral ischemic lesions than rate control strategies.^8–10^ However, the optimal rhythm control option in CF patients is still of concern, as the majority of anti-arrhythmic drugs carry a high risk of adverse events like pro-arrhythmias and negative inotropic effects, potentially worsening heart failure; as such, only amiodarone is typically proposed, although its use is accompanied by extracardiac adverse effects.^11–14^

In this setting, catheter ablation of AF has emerged as a safe and effective alternative for rhythm control, even among CHF patients. Despite its clear efficacy for symptomatic AF patients, its role within CHF patients is less well defined.^14^ Small randomized trials and observational studies, as well as a large collaborative meta-analysis encompassing up to 1,800 patients, have assessed the role of AF ablation in CHF patients. The optimal patient selection, timing referral and ablation strategy remain, however, unclear, especially among those individuals with specific cardiomyopathies underlying CHF. The present systematic review aims to discuss patients’ selection, safety, efficacy and clinical implications of AF catheter ablation in the setting of CHF and specific cardiomyopathies.

## Methods

A systematic review was conducted to retrieve all published data concerning AF ablation in patients with CHF. MEDLINE/PubMed and the Cochrane database were searched for pertinent articles published in English from 2002 to December 2016, according to published recommendtions.^15^ The terms “atrial fibrillation” AND “catheter ablation” AND “heart failure” AND (“clinical trial” OR “meta-analysis” OR “observational study”) were used to identify all of the published articles referring to this specific patient population. Moreover, a second search was performed to identify published data concerning catheter ablation of AF (AFCA) in patients with specific structural cardiomyopathies. The terms “atrial fibrillation” AND “catheter ablation” AND (“cardiomyopathy” OR “valvular”) AND (“clinical trial” OR “meta-analysis” OR “observational study”) were used.

If the citations were deemed potentially pertinent, they were appraised as complete reports according to the following selection criteria: 1) human studies; 2) published between 2002 and 2016; and 3) investigating patients with impaired LV systolic function, defined as LV ejection fraction (LVEF) <50%, or who had specific cardiomyopathies, undergoing AF transcatheter ablation. Exclusion criteria were: 1) non-human setting; 2) duplicate reporting (in which case, the manuscript reporting on the largest sample of patients was selected); and 3) studies including patients undergoing surgical or hybrid AF ablation.

## Search results

The first search identified 176 abstracts; among this group, 149 were excluded following application of the inclusion and exclusion criteria. Ultimately, 27 studies were finally selected and included, including in particular 19 observational studies, four randomized controlled trials (RCTs), and four meta-analyses.^16–42^ Details concerning sample size and main findings for each of the studies considered are summarized in **Tables 1 and 2**. The second search identified 52 abstracts; among this group, 38 were excluded following application of the inclusion and exclusion criteria. Fourteen studies were finally selected, with one study in particular including patients with tachycardiomyopathy (TCM), eight studies including patients with hypertrophic cardiomyopathy (HCM), and five studies including patients with valvular cardiomyopathy.^43–56^

Observational studies. As detailed in **Table 1**, observational studies included 1,504 patients. Follow-up ranged from six to 72 months. The mean efficacy of AF ablation in maintaining sinus rhythm (SR) was 45% after the first procedure, rising to 73% upon inclusion of redo procedures. Rate of complications was 4.2%. Several studies reported on improvement in LV systolic function, quality of life, exercise tolerance and functional class, mitral regurgitation, reduction of heart failure hospitalizations and incidence of stroke.^16–19,22–28,31–33^

Two studies selectively focused on patients with TCM, specifically 113 individuals. AF ablation efficacy at follow-up (six to 18 months) was 74%, and mean LVEF significantly improved from 35% or 40% to 54%, suggesting that TCM did not increase the risk of AF recurrence.^27,43^

Randomized controlled trials. The four available RCTs included 115 patients overall **(Table 1)**. The control groups included patients undergoing atrioventricular node ablation and biventricular pacemaker implantation in the trial by Khan et al.,^35^ and patients being treated with optimal medical therapy for rate control in the other three trials. Follow-up ranged from six to 10 months. The mean efficacy of AFCA in maintaining SR was 59% after the first procedure, rising to 77% after including patients undergoing repeat procedures. The observed complication rate was 21%. Three of the four studies also found improvements in quality of life and function, measured via a six-minute walking test (6MWT) and peak *V*O_2_ at cardiopulmonary exercise test, respectively.

**Figure 1:**
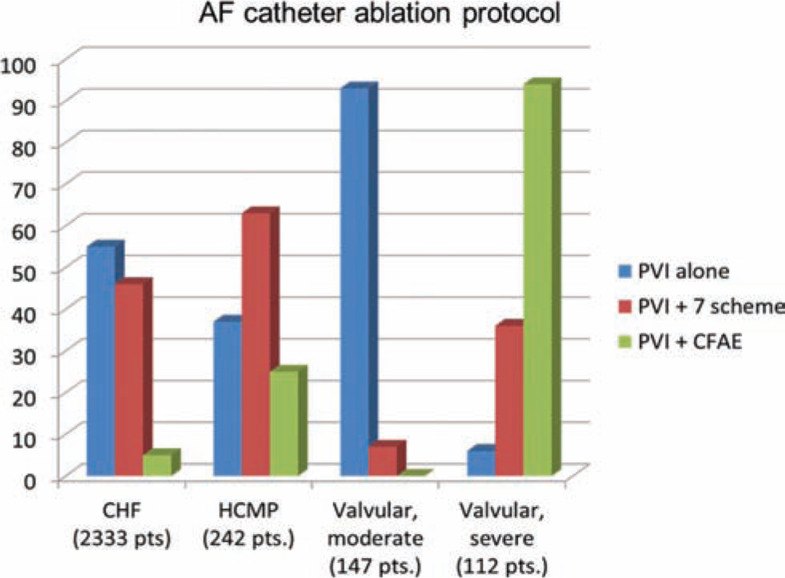
AF catheter ablation protocol according to underlying cardiomyopathies. AF: atrial fibrillation; CHF: chronic heart failure; HCMP: hypertrophic cardiomyopathy; CFAE: complex fractionated atrial electrograms.

AF ablation in specific cardiomyopathies and valvular heart disease. As listed in **Table 2**, eight studies described the outcome of AF ablation in HCM, comprised of 242 patients. The mean efficacy following a first ablation procedure was 46%, improving to 71% with repeated procedures (with a follow-up range of six to 40 months.) Of note, the majority of these studies approached AF via extensive left atrial ablation including PV isolation, linear lesions and complex fractionated atrial electrogram (CFAE) ablation **(Figure 1)**.

**Figure 2:**
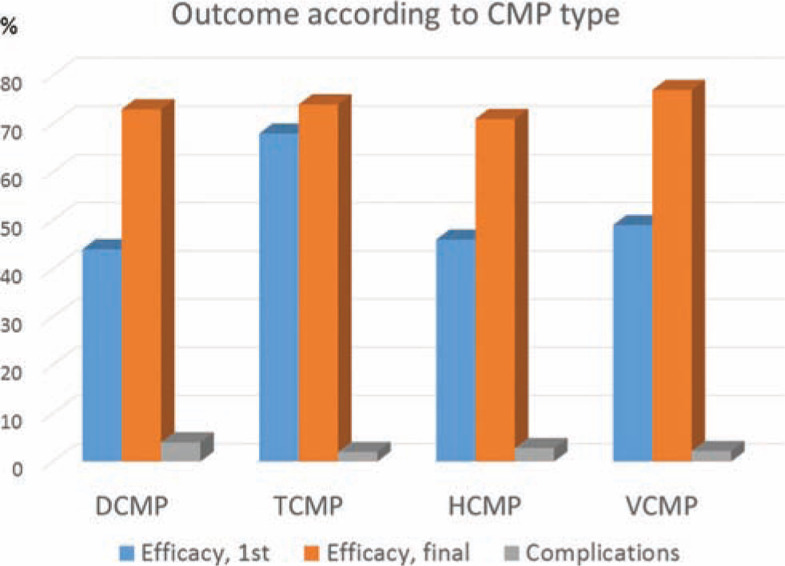
Overall outcome, including success after single and after last procedure, and complications stratified according to the underlying cardiomyopathy. CMP: cardiomyopathy; DCMP: dilated cardiomyopathy; HCMP: hypertrophic cardiomyopathy; TCMP: tachycardia-induced cardiomyopathy; VCMP: valvular cardiomyopathy.

Five studies reported the outcome of AF ablation in patients with significant valvular cardiomyopathy, defined as at least moderate mitral or aortic regurgitation or stenosis, or previous valvular surgery. These studies included 259 patients followed for 11 to 54 months. Mean efficacy following the first ablation procedure was 49%, improving to 77% after the over 40% of patients undergoing repeated procedures were included **(Figure 2)**.

## Discussion

AF ablation in CHF patients remains a still-growing body of knowledge. At present, the majority of available data is based on small observational single-center studies, predominantly of a retrospective nature. Overall, AF ablation reported positive results in terms of both safety and efficacy in maintaining SR, comparable to patients without CHF. Additionally, LV function shows a significant improvement in these patients during follow-up **(Table 3)**.

Concerning ablation protocol, the mainstay of the procedure is PV isolation for all of the patients; additionally, more than half of the patients, according to the current knowledge and available tools, underwent additional linear lesions (e.g. the “seven scheme,” a lesion set including, in addition to PV isolation, a roof line connecting superior PVs and a mitral isthmus line connecting the left inferior PV to the mitral annulus; or CFAE ablation).^57–59^

A relatively large number of repeated procedures (i.e. about one-third of patients) are described. In general, the advanced structural and electrical atrial remodeling characteristic of these patients seems not to impact the final outcome of ablation, although it is frequently associated with the need for multiple procedures to maintain SR.^4^ Concerning long-term follow-up, six studies focused on late outcome (i.e., of more than two years) following AF ablation, finding that despite lower efficacy after a single procedure (around 30–50%), the overall efficacy including repeated procedures was ultimately higher, at 70% to 80%.^21,26,28,31–33^ Interestingly, despite more procedures being performed per patient in these cases, the complication rates were similar to those in previous studies, mainly due to the use of improved technologies and procedural amendments, such as performing ablation under uninterrupted anticoagulation.

All of the studies consistently reported a significant improvement in LV systolic function following AF ablation, measured by echocardiographic LVEF (mean improvement from baseline to follow-up end = +13%) This finding is not surprising, as AF ablation holds the potential to interrupt the vicious circle that leads to LVEF reduction following AF.^5^

Several studies reported improvements in quality of life, symptom reduction and/or functional class improvement following AF ablation.^16–18^ Additionally, Ullah et al. reported lower incidences of stroke and death among patients in SR following AF ablation, while Bunch et al. reported long-term reductions in mortality and hospitalization for heart failure following the performance of ablation, compared with outcomes with medical therapy; this finding in particular warrants further attention and testing in prospective studies.^31,34^ In fact, similar findings were also more recently reported by Di Biase et al., in terms of mortality reduction with ablation as compared with the use of amiodarone for the treatment of CHF patients.^60^

Overall, four short-term RCTs have been performed on a limited population. These studies confirmed the safety and efficacy of the procedure, except for MacDonald et al., who reported lower success rates and no occurrence of improvement in LVEF or exercise tolerance.^36^ However, it should be noted that patients included in this study had advanced CHF, longer AF duration and/ or a worse functional class than those in the other RCTs. Additionally, the complication rate in this study was higher than those in the majority of observational studies, likely because a larger proportion of patients with advanced CHF were included, and a higher prevalence of extensive LA or biatrial ablation was performed, necessitating longer procedural times and a higher amount of fluid administration, and carrying a more significant risk for post-procedure complications.

Additionally, a large multicenter, collaborative meta-analysis including more than 1,800 patients over a mean follow-up period of two years demonstrated a similar improvement in LVEF, while safety and efficacy were similar to data from the general population.^41,61–63^ This study additionally focused on the reduction of the proportion of patients with severely depressed LV function. Its findings, previously reported by a single-center experience, is of paramount clinical importance since they potentially suggest that AF ablation, on top of optimal medical treatment, has the potential to reduce the proportion of CHF patients requiring implantation of cardioverter-defibrillators for the primary prevention of sudden death.^64^ Of note, time to first AF diagnosis and CHF diagnosis significantly correlated with degree of success following ablation outcome, highlighting the importance of prompt optimal treatment of both CHF and AF to achieve the best clinical results.

Eventually, one small observational prospective study was conducted that specifically investigated patients with CHF and preserved LVEF undergoing AF ablation.^32^ This study, including 74 patients, reported a low (27%) efficacy after the first procedure that increased to 73% when including repeated procedures and antiarrhythmic drugs (with a follow-up of 43 months). Of note, LV diastolic and systolic function measured by echocardio-graphic strain and strain rate improved only in patients maintaining stable SR.

Two studies specifically focused on patients with TCM, and showed that TCM itself relates to good outcome following AF catheter ablation, even after the first ablation procedure.^27,43^ The same finding was reported in a long-term follow-up sub-analysis by Anselmino et al.,^26^ highlighting the benefits of AF ablation in this population subset.

AF catheter ablation in the setting of “difficult” cardiomyopathies. HCM is related to an increased incidence of AF, but rhythm control strategies frequently obtain poor results. Concerning AF ablation outcome, eight observational studies have been conducted among HCM patients.^44–51^ Consistently, all studies reported a very limited efficacy after a single ablation procedure. However, about half of the patients underwent repeated procedures, raising the efficacy up to 70% to 80%; in this respect, the prevalence of extensive LA or biatrial ablation, including linear lesions and CFAEs, was significantly higher as compared with more “classical” CHF patients **(Figure 1)**. This finding reflects the difficulties in achieving effective rhythm control: patients with HCM present a complex substrate, characterized by severe left atrial enlargement, fibrosis, and structural and electrical negative remodeling that impacts the outcome following AF ablation.^65^ However, rhythm control warrants careful consideration, as AF worsens the long-term outcome of these patients with respect to both quality of life and prognosis.^66^

Another “difficult” setting is in patients with significant valvular cardiomyopathies; in particular, mitral valve disease. Five studies reported on the outcomes of AF ablation among patients with significant valvular disease. Three of them, which included patients with prior cardiac valvular surgery or previous percutaneous interventions for mitral rheumatic stenosis, reported a very limited efficacy after a single procedure that increased to 70% when repeat procedures—more than half of the total—were included.^53,55,56^ In fact, the peculiar electroanatomical atrial substrate determined by rheumatic heart disease is characterized by profound structural remodeling, extensive fibrosis and collagen replacement, requiring consequently extreme substrate modification to achieve stable SR.^67,68^

Of note, the prevalence of persistent AF among the included population was relatively high, and this may have influenced the considerable prevalence of LA extensive ablation protocols. Indeed, CHF patients, and even more so patients with HCM or valvular disease, present significant structural remodeling, resulting in a higher risk of persistent AF development as compared with “lone” AF.

Aiming to improve the outcome of persistent AF ablation, rotors (areas of micro re-entries) and focal sources of high-frequency activity have been proposed as theoretically pivotal points for AF perpetuation and therefore, targets for ablation.^69^ However, of note, among non-selected AF patients, this approach showed no benefit, but did demonstrate longer procedural times and a higher risk of complications.^70^ These results, along with those derived from the general population concerning use of linear lesions and CFAE in persistent AF ablation, underline the limited efficacy of a traditional ablation approach, including PV isolation alone, and also the limited efficacy of standard approaches in this population setting.^71^

Therefore, as for persistent AF, research should be directed towards achieving a better understanding of AF pathogenesis in cases of advanced atrial substrate remodeling, which may eventually result in better outcomes following ablation. In fact, the optimal approach to patients with advanced atrial remodeling, such as those with long-standing persistent AF, valvular disease, and/or HCM, still needs to be defined.

## Conclusions

## Clinical implications and future perspectives

Following the above-mentioned evidence, AF catheter ablation can be considered to be a safe procedure that presents low complication rates even in patients with complex atrial substrates and/or comorbidities, such as those with CHF. Technological innovations contribute to improve its safety: the use of superirrigated catheters leads to a significant reduction in fluid administration during ablation, and contact force sensing enables for better optimization of radiofrequency delivery and titration.^72,73^ Moreover, magnetic resonance or computer tomography imaging can correctly define patients’ anatomy, enabling more precise mapping of the atrial area and PVs in order to pre-define ablation protocol.^74,75^ However, this data refers predominantly to high-volume centers: because of the complexity of such patients with CHF, the suggestion is for the referral of them to experienced, larger centers more skilled in and capable of managing plausible complications. For example, performing the procedure on anticoagulants minimizes the risk of clinical and asymptomatic thromboembolic complications in the general population, and this should be considered even in the CHF subset, who often require longer procedural times. Additionally, radiation exposure reduction, favored by fluoro-scopy-zero mapping technologies, is also warranted.^76^

Additionally, AF ablation improves LV function over the short-and long-term follow-up, especially when compared with the effects of medical treatment. This finding is not surprising: the interruption of the vicious circle between AF and CHF, the restoration of regular cardiac cycles and normal atrial mechanical function holds the potential to slow the negative electrical and structural remodeling of the failing heart, leading to significant clinical benefits.^5,77^ Consequently, AF ablation drives towards a significant improvement in quality of life, functional class and exercise capacity.

In general, the shorter the history of both CHF and AF is, the better the outcome is; so, a shorter AF and CHF history and a milder LA dilation are plausible markers of favorable outcome. The absence of signs of advanced myocardial disease, such as fibrosis at magnetic resonance imaging, is likely related to a significant improvement in LV function. Conversely, patients with advanced CHF, unstable hemodynamic conditions and/or poor functional class are more prone to complications and are less likely to take advantage from AF ablation; in this setting, ablation should not be proposed as a means to improve symptoms or prognosis.

Concerning the ideal ablation protocol among CHF patients, PV isolation alone seems to be sufficient for selected patients, such as those with paroxysmal AF, a short AF and CHF history and mild LA dilation, at least for the first procedure. Conversely, patients with a long history of both CHF and AF as well as severe LA dilation possibly require a more distinct ablation approach, including targeting of non-PV mechanism to warrant stable SR. However, at present, both technological and intellectual improvements are needed that aim to define the optimal approach to patients with advanced atrial remodeling. Additionally, in the setting of specific, high risk subset populations, such as those with HCM and/or severe valvular cardiomyopathies, left atrial substrate modification should probably be considered as first-line to maintain SR. However, these considerations about ablation protocols need to be tested in prospective randomized trials on CHF patients. Most of all, the impact of AF ablation on hard outcomes, such as mortality and stroke incidence, still needs to be tested in prospective, randomized trials.

## Figures and Tables

**Table 1:** Observational Studies and Randomized Trials Focusing on AF Transcatheter Ablation in CHF Patients

First Author, Year (ref.)	Number of Patients	Age, Years	Paroxysmal AF (%)	NYHA Class	Final Results						
					Complications (%)	FU, Months	Success Single (%)	Redo (%)	Success Final (%)	LVEF (%)	Other Parameters
***Observational Studies***											
Chen 2004^16^	94	57	51	2.8	4	14	52	22	73	36→41	↑QoL.
Hsu 2004^17^	58	56	9	2.3	3	12	28	50	78	35→56	↑LVD, QoL, exercise capacity and NYHA.
Tondo 2006^18^	40	57	25	2.8	13	14	55	33	87	33→47	↑Exercise capacity and QoL.
Gentlesk 2007^19^	67	54	70	–	–	6	55	31	86	42→56	–
Efremidis 2007^20^	13	54	0	–	0	9	62	–	62	36→52	–
Nademanee 2008^21^	129	67	40	–	4	27	–	21	79	30→37	–
Lutomsky 2008^22^	18	–	100	–	–	6	50	–	–	41→52	–
De Potter 2010^23^	36	52	39	–	5	16	50	31	69	41→58	–
Choi 2010^24^	15	56	67	1.7	6	16	47	27	73	37→50	–
Cha 2011^25^	111	55	28	–	3	12	–	–	76	35→56	↑QoL.
Anselmino 2013^26^	196	60	22	2.1	5	46	45	30	62	40→50	↑NYHA and mitral regurgitation.
Calvo 2013^27^	36	52	24	–	5	6	70	31	83	41→48	–
Nedios 2014^28^	69	61	33	2.4	1	28	40	46	65	33→48	–
Kosiuk 2014^29^	73	59	32	–	–	12	37	30	–	37→41	↓ ICD shocks.
Lobo 2014^30^	31	60	7	2.2	3	20	–	26	77	44→59	–
Bunch 2015^31^	267	66	–	–	–	60	39	–	–	27→42	↓ Death and CHF hospitalization vs. AF; no ablation.
Rillig 2015^33^	80	60	20	2.0	1	72	35	–	57	35→56	↑NYHA, better outcome for TCM.
Ullah 2016^34^	171	58	36	2.3	5	42	26	60	65	34→46	↑NYHA, tstroke and death in AF recurrence.
Overall	1504	58	21	2.3	4	35	44	37	73	+ 13%	–
***AF Ablation in Patients with Tachycardiomyopathy***											
Calvo 2013^27^	61	52	22	–	8	6	73	–	80	40→54	TCM doesn't relate to AF recurrence.
Sairaku 2014^43^	52	61	0	–	0	18	–	–	67	35→54	↑ LVEF improvement in patients in SR; TCM doesn't relate to AF recurrence.
Overall	113	56	11	–	4	12	68	–	74	+ 16%	
***Randomized Controlled Trials***											
Khan 2008^35^	41	60	49	–	12	6	71	20	88	27→35	↑QoL and 6MWT distance vs. AV node ablation.
MacDonald 2010^36^	22	62	0	2.9	15	10	–	30	50	36→41	QoL and 6MWT: no difference vs. medical treatment.
Jones 2013^37^	26	64	0	2.4	11	10	69	19	88	21→32	↑QoL and peak *V*O_2_, ↓ BNP vs. rate control.
Hunter 2014^38^	26	55	0	2.7	5	6	38	54	81	32→40	↑QoL, NYHA class peak *V*O_2_, ↓ BNP vs. rate control.
Overall	115	60	12	2.7	10	8	59	31	77	+ 8%	

CHF: chronic heart failure; AF: atrial fibrillation; FU: follow-up; LVEF: left ventricular ejection fraction; QoL: quality of life; LVD: left ventricular diameter; ICD: implantable cardioverter-defibrillator; 6MWT: 6-minute walking test; TCM: tachycardiomyopathy; SR: sinus rhythm.

**Table 2:** Observational Studies Concerning AF Catheter Ablation in Specific Subset Cardiomyopathies

Author and Year (valvular 1 cardiomyopathy subtype)	Number of patients	Age, Years	Paroxysmal AF (%)	NYHA Class	Follow-up, Months	Success Single (%)	Success Final (%)	Procedural Characteristics C	Complications (%)
***Hypertrophic Cardiomyopathy***									
Liu 2005^44^	4	57	100	2.0	6	75	100	PVI.	0
Kilicaslan 2006^45^	27	55	52	–	12	52	70	PVI.	0
Di Donna 2010^45^	61	54	57	2.0	29	28	67	PVI + 7 scheme.	0
Bunch 2008^47^	33	51	64	–	30	–	74	24% PVI; 76% PVI + 7 scheme.	12
Derejko 2013^48^	30	49	47	1.9	12	33	53	42% PVI; 58% PVI + 7 scheme + CFAE.	0
Santangeli 2013^49^	43	59	28	1.8	15	49	94	PVI + 7 scheme + CFAE.	0
Mussigbrodt 2014^50^	22	57	45	–	–	41	54	68% PVI; 32% PVI + 7 scheme.	5
Okamatsu 2014^51^	22	65	23	–	21	45	59	PVI.	
Overall	242	56	52	1.9	18	46	71	28% PVI; 72% PVI + substrate.	2
***Valvular Cardiomyopathies***									
Khaykin 2004^52^ (moderate mitral or aortic stenosis or regurgitation)	102	64	37	1.4	11	83	93	PVI.	3
Wang 2009^53^ (Mitral or aortic prosthetic valves or previous mitral commissurotomy)	51	48	0	–	12	51	67	PVI + CFAE.	2
Miyazaki 2010^54^ (moderate mitral or aortic stenosis or	45	66	80	1.3	26	47	78	80% PVI; 20% PVI + 7 scheme.	4.3
regurgitation) Gu 20 1 0^55^ (Rheumatic heart disease six months after valvular surgery)	47	55	0	–	54	32	79	57% PVI + CFAE + 7 scheme; 33% PVI + CFAE; 10% PVI alone.	4
Derejko 20 1 4^56^ (Previous mitral valve surgery or percutaneous mitral commissurotomy)	14	55	29	–	23	36	71	93% PVI + CFAE + 7 scheme; 7% PVI alone.	0
Overall	259	58	29	1.4	25	49	77	56% PVI alone; 44% PVI + substrate.	2

AFCA: atrial fibrillation catheter ablation; PVI: pulmonary vein isolation; CFAE: complex fractioned atrial electrogram.

**Table 3:** Main Results Concerning Safety, Efficacy, and Other Relevant Outcomes Stratified by Type of Underlying Cardiomyopathy

Type of Cardiomyopathy	Number of Patients	First Procedure Success (%)	Final Success (%)	Complications (%)	Comments
DCMP	1,619	45	73	4.2	LVEF improvement +12%. NYHA/6MWT improvement.
TCMP	113	68	74	4.0	LVEF improvement +16%. Higher first procedure success.
HCMP	242	46	71	2.8	High prevalence of CFAE/lines and redo procedure. NYHA improvement.
VCMP	259	49	77	2.2	High prevalence of CFAE/lines and redo procedure.

DCMP: dilated cardiomyopathy; TCMP: tachycardia-induced cardiomyopathy; HCMP: hypertrophic cardiomyopathy; VCMP: valvular cardiomyopathy; LVEF: left ventricular ejection fraction; CFAE: complex fractionated atrial electrogram; NYHA: New York Heart Association; 6MWT: six-minute walking text.
